# Following the Science? Examining the Issuance of Stay-At-Home Orders Related to COVID-19 by U.S. Governors

**DOI:** 10.1177/1532673X221106933

**Published:** 2022-06-10

**Authors:** Gregg R. Murray, Susan M. Murray

**Affiliations:** 1421 Augusta University, Augusta, GA, USA

**Keywords:** COVID-19, public health, policy diffusion, executive orders, governors

## Abstract

Informed by the public health policymaking literature, this study’s objective is to identify scientific, political, social, economic, and external factors related to U.S. governors’ decisions to issue stay-at-home orders (SAHOs) in response to the first wave of the COVID-19 pandemic. Public health experts advocate for social distancing to slow the spread of infectious diseases, but government mandates to social distance can impose substantial social and economic costs. This study uses event history analysis to investigate the issuance of COVID-19-related gubernatorial SAHOs during a 41-day period in the 50 U.S. states. The findings indicate that scientific, political, and economic factors were associated with the issuance of SAHOs, but that external considerations played the largest role, particularly those related to the timing of other governors’ decisions. This study offers evidence about how some U.S. political leaders balance public health concerns against other considerations and, more broadly, how state governments address crisis-level issues.

## Introduction

Following urgent warnings from public health experts, there was a surge of new policies as governments around the world took dramatic steps to limit the spread of the first wave of the coronavirus disease 2019 (COVID-19) (Weible et al., 2020). In an effort to force social distancing, a primary tool used to stop the spread of infectious diseases, government authorities in the U.S. incrementally restricted social contact starting with prohibiting large gatherings of people, closing schools, and limiting or closing non-essential businesses ultimately culminating in comprehensive, statewide stay-at-home orders (SAHOs) in a large majority of U.S. states. Though specific restrictions vary from jurisdiction to jurisdiction, SAHOs, which are also referred to as “shelter-in-place” orders, require people to remain in their residences except to shop for grocery and pharmacy items, seek medical care, or work in essential businesses. While SAHOs protect public health and safety, they impose substantial social and economic costs on society (e.g., Curley & Federman, 2020; Gostin & Wiley, 2020; Pew Research Center, 2020). For instance, in the case of COVID-19 in the U.S., basic constitutional rights such as freedom of assembly and free exercise of religion were curtailed (Henson, 2021). Further, over 55 million children were prevented from attending school (Camera, 2020), more than 20 million people filed initial jobless claims over a 4-week period (J.P. Morgan, 2020), and **almost 300 million Americans**, about 90% of the country, were required to remain in their residences for several weeks (Norwood, 2020).

Given the profound effects and high-stakes nature of these SAHOs, the objective of this study is to identify factors related to U.S. governors’ decisions to issue a coronavirus-related stay-at-home order or not in their states in response to the first wave of the disease in early 2020. In particular, this study evaluates potential public health-related scientific factors, the primary drivers one would expect to influence public health policy, and other potential factors related to political, social, and economic conditions as suggested in the public health policymaking literature (e.g., Brownson et al., 2009; Spasoff, 1999), as well as factors external to a state that may have affected these decisions (Berry and Berry, 1992; Mooney & Lee., 1995). The next section of this manuscript presents background information on the incremental policy responses followed by pertinent literature regarding the potential explanatory factors and resulting expectations. The following section describes the event history analysis method used and the novel dataset analyzed. The results section, which follows, suggests that governors’ decisions regarding SAHOs were influenced by scientific, political, economic, and, particularly, external considerations but not social considerations. The final section summarizes the findings, notes the study’s limitations, and puts this research in a broader context about leaders’ decisions during public health crises and, more broadly, crises in general.

### Background

Beyond the ability to dominate communications during crises, the U.S. federal government holds limited health-related powers, which are chiefly derived from regulatory powers related to interstate and foreign commerce (National Conference of State Legislatures, 2014). In the U.S., states are primarily responsible for public health based on authority derived from police powers granted to states by the 10^th^ Amendment to the U.S. Constitution (National Conference of State Legislatures, 2014). State public health powers are primarily exercised by governors, and governors visibly use their unilateral executive powers to pursue public health objectives. These objectives often include addressing health emergencies, establishing new public health programs, directing and reorganizing health agencies, and increasing the profile of public health issues (Gakh et al., 2013). For instance, governors have issued executive orders to permit out-of-state emergency medical technicians to respond to disasters (e.g., Louisiana Executive Order No. BJ 2010-9, 2010), create state health insurance exchanges in response to healthcare reform (National Conference of State Legislatures, 2018), and establish prescription drug monitoring programs (e.g., Missouri Executive Order 17-18, 2017). These orders are particularly likely when legislation, regulation, or litigation is insufficient or unavailable in a timely fashion (Gakh et al., 2013). Given the large number of governors who issued coronavirus-related SAHOs (42 out of 50 or 84.0%) in a short period of time (19 days; Mervosh et al., 2020), it is easy to conclude that governors primarily focused on the health emergency aspects of the events and not political, social, or economic aspects.

Governors took a number of incremental, non-pharmaceutical steps to address the coronavirus outbreak before taking the dramatic step of ordering people to stay at home (for details see Kaiser Family Foundation, 2020). In most cases, governors first declared states of emergency (see Fowler, Kettler, and Witt [2021] for further details) as the virus began to spread and coronavirus-related deaths increased. The governor of Washington State led by declaring a statewide state of emergency on February 29, the day of the first confirmed death due to COVID-19 on U.S. soil in Kirkland, Washington. All the other governors quickly followed suit with the governor of West Virginia, the last state, declaring a state of emergency on March 16. Emergency declarations free up resources and waive legal constraints, both state and national, on state actions but place few restrictions on social or economic activities.

The next increment of orders related to slowing the spread of the virus in order to not exceed state healthcare capacity to treat infected patients, the so-called “flatten-the-curve” declarations. To comply with CDC (Center for Disease Control and Prevention) recommendations to start “social distancing,” or staying physically separated at a safe distance from other people, government agencies at the state and local levels began restricting social gatherings. The first state-level “gathering” restriction, for indoor groups of 100 or people, was put in place on March 10 by the governor of Ohio. This was followed the next day by similar restrictions in California, Kentucky, Rhode Island, and Washington. By March 26, all 50 states had issued gathering restrictions.

Governors followed next with more disruptive orders that closed schools on a statewide basis starting on March 16 in Maryland, Michigan, Ohio, and Oregon. Eventually, all 130,000 of the nation’s schools were closed. Restrictions for the country’s one million restaurants began in some states on March 15, followed on March 17 by closures of other non-essential businesses in many states. This incremental process of restrictions then closures was generally followed by the issuance of statewide stay-at-home orders, the first of which was issued by California on March 19. By April 7, 42 of the 50 governors had issued blanket SAHOs allowing only “essential” activities (as opposed to targeted SAHOs that were, for instance, age restricted), with the governors of Arkansas, Iowa, Nebraska, North Dakota, Oklahoma, South Dakota, Utah, and Wyoming deciding not to issue a statewide SAHO.

Though the specifics vary from state to state, stay-at-home orders mandate people stay in their homes except to shop for grocery and pharmacy items, seek medical care, or work in essential businesses. Many of the SAHOs indicate that compliance is enforced through civil or criminal penalties such as fines of up to several thousand dollars, orders to suspend business operations, and arrest or imprisonment. News reports indicate individuals in Illinois, Maryland, and New Jersey were cited, charged, or arrested for violating coronavirus-related SAHOs (Norwood, 2020).

### A theoretical framework, public health policymaking factors, and expected effects

In most states, the authority to issue a public health-related SAHO rests with the executive branch, the governors, and there is significant variance in the timing of governors’ issuance of such orders. Public policy researchers have identified two broad factors that affect the adoption of governmental policies by states: internal state determinants and external diffusion from other states (Berry and Berry, 1999; Mooney & Lee., 1995). Public health scholars have categorized internal determinants, factors that make a policy more or less appealing to a state’s policymakers, as scientific, political, social, and economic factors (Brownson et al., 2009; Spasoff, 1999). External effects, on the other hand, refer to the influence on a state’s policymakers by the policy choices of other states, such as, geographically proximate neighbors and temporal adoption patterns of other states (Berry & Berry, 1992).

Scientific evidence has informed public health policy to great effect in domains such as preventing the spread of infectious diseases with vaccinations, slowing addiction with tobacco control, and controlling pollution with clean air and water regulations (Brownson et al., 2017: xiii). The *scientific factors* affecting the adoption of SAHOs may include considerations regarding “flattening the curve” and population vulnerability. Efforts to prevent or delay an overwhelming increase in cases are commonly referred to as “flattening the curve” (Centers for Disease Control (CDC) and Prevention, 2007). The healthcare systems in states with more incidents of a disease may be more likely to be overwhelmed with patients, in extreme cases leading to more serious healthcare outcomes including deaths. Social distancing is a primary tool used to stop the spread of infectious diseases (CDC, 2020a) and flatten the curve, and SAHOs are a powerful tool government authorities have to mandate social distancing. This suggests that *governors in states with greater prevalence of the disease in relation to healthcare capacity will be more likely to issue a SAHO*.

Some populations, such as older adults and people with serious underlying medical conditions, are more vulnerable to the disease than others (CDC, 2020b). The health of residents from state to state varies considerably (Kaiser Family Foundation, 2019). As such, the residents of some states may be more vulnerable to the coronavirus than residents of other states. Some scholars have argued that states are obligated based on the social contract to protect their citizens from predation, which can include pathogenic predation, and failure to do so risks governmental de-legitimation (Price-SmithAndrew, 2009). This suggests that *governors with more vulnerable populations will be more likely to issue a SAHO*.

Some argue that political actors – public office holders, civil servants, and interest groups – are “the most important players” in public health policy (Spasoff, 1999: 6). For instance, Karol and Thurston (2020) argue that starting in the mid-1980s, political partisanship began driving California state legislators’ vote on abortion policy. As conceptualized in this research, the *political factors* affecting the issuance of SAHOs include considerations regarding leader and electorate partisanship. Stay-at-home orders are unilateral gubernatorial executive orders that in most cases are equivalent to enacted legislation (e.g., Sellers, 2017) and typically go unchecked by the other branches of government (Cockerham & Crew, 2017). Broadly, political liberals tend to support greater government intervention in society to address society’s challenges, while political conservatives tend to favor larger roles for individuals, private institutions, and markets. Although the correspondence between ideology and partisanship is not perfect, Democrats typically embrace a liberal political belief system and Republicans a conservative one (Lewis-Beck et al., 2008). Further, evidence suggests that party interests are playing a larger role in gubernatorial behavior (Ferraiolo, 2017; Fowler et al., 2021; Jensen, 2017), and that, indeed, American political behavior has become nationalized (Hopkins, 2018). Hopkins suggests that national politics convey evocative information to voters that conjures up consequential associations with national parties, the issues they promote or oppose, and the groups they support or oppose. Because this information may be particularly salient in low-information contexts or situations of great uncertainty, such as the early days of the pandemic, partisan cues from national leaders, particularly the president, may affect a governor’s decision to issue a SAHO, whether the governor is Democratic or Republican (e.g., Butler et al., 2017). In the case of coronavirus in the U.S. in 2020, the Republican president who saw the disease as less threatening may have influenced Republican governors to delay or decline to issue a SAHO, while Democratic governors may have reacted to the limited response of a Republican president and acted more quickly (e.g., McCann, Shipan, and Volden, 2015). Early evidence (from March 2020) from Baccini and Brodeur (2021) shows Democratic governors were 50% more likely than Republican governors to issue a stay-at-home order. This suggests that *Democratic governors will be more likely to issue COVID-19-related SAHOs than Republican governors*.

People’s vote choices are heavily influenced by their partisanship: Democrats favor Democratic candidates and Republicans favor Republican candidates (Lewis-Beck et al., 2008). Research indicates the H1N1 virus (swine flu) crisis in 2009 polarized public attitudes in the U.S. along party lines such that Democrats were more responsive to pro-vaccination messages than Republicans (Baum, 2011). In the case of the 2019 coronavirus, early public opinion polling showed a similar partisan divide, with Democrats being more supportive of taking steps to slow the spread of the disease (94%) than Republicans (68%; Kaiser Family Foundation, 2020). Evidence also indicates voters hold governors accountable for policy outcomes (e.g., Niemi et al., 1995), and elected officials, like governors, are strongly, if not primarily, motivated to win re-election (e.g., Mayhew, 1974). This suggests that candidates for office in states with more Democratic voters would fare better at the polls when they favor more government action in response to a public health crisis than when they favor less government action. Consequently, it is reasonable to conclude that *governors with an electorate with a higher percentage of Democrats will be more likely to issue a COVID-19-related SAHO*.

Karol and Thurston (2020) also argue that prior to the mid-1980s ascension of political considerations, that social factors, in the form of legislators’ religious values, drove California state legislators’ votes on abortion policy. Among more broadly based social considerations are attitudes about sacrificing for the public good. Countries have long been categorized as having collectivistic versus individualistic cultures, and Elazar (1966) extends this to U.S. states. He contends that the civil society of states can be categorized in terms of how they prioritize individual versus community interests, arguing, for instance, that “individualistic” and “moralistic” states in particular seek to promote private interests relative to community interests. Other scholars have focused specifically on an individualism-collectivism dimension. Generally speaking, individualism focuses on promoting individual autonomy and independence, while collectivism focuses on group membership and interdependence (Vandello & Cohen, 1999). In this research, the *social factor* affecting the adoption of SAHOs may include considerations regarding civic mindedness. Citizens of more collectivistic states will be more willing to make costly sacrifices for others than citizens in states with more individualistic civil societies. Social distancing requires significant social and financial sacrifices by individuals on behalf of their communities to slow the spread of a disease through measures such as closing schools, businesses, and places of worship. This suggests that *governors in states with a more collectivistic civil society will be more likely to issue a SAHO*, because their citizens will be more likely to tolerate the social and economic costs.

Protecting the public’s health is an expensive endeavor, so economic considerations also play an important role in the formulation of public health policy. Teutsch et al. (2016) note that the U.S. Congressional Budget Office and the Office of Management and Budget use cost estimates to score proposed policies and evaluate their costs and benefits across a number of dimensions including economic burden. As conceptualized in this study, the *economic factor* affecting the adoption of SAHOs includes considerations regarding states’ economic health. A governor who issues a SAHO is likely to understand that the order will negatively and substantially impact the state’s economy at least over the short term (Correia et al., 2020).^1^ Governors in states with stronger economies may calculate that their economy can weather restricted activity in the short run, while governors in states with weaker economies may estimate that they have less latitude in this regard. This argument suggests that *governors in states with a stronger economy will be more likely to issue a COVID-19 SAHO*.

Finally, turning from internal to *external factors* affecting the issuance of SAHOs, policy researchers also consider geographic and temporal diffusion. A number of studies show that states are influenced by states with shared borders, a phenomenon often referred to as geographic diffusion (e.g., Chamberlain & Haider-Markel, 2005), and this influence is motivated by learning, imitation, cooperation, and competition (e.g., Berry, 1999). In particular, being informed by the experiences of neighboring states, with their similarities in media, populations, and common values (Mooney, 2001), assists states in reducing the risks inherent in making policy. A governor may imitate another governor whose approach may be deemed “an appropriate response to a given problem” or whom may “give cover” in the case of consequential measures such as SAHOs (Maggetti & Gilardi, 2016). Conversely, a governor may want to gain a competitive advantage for her or his state such as keeping the state’s economy open to attract business from states in which the economy has been closed down (Berry & Berry, 1992). Further, and from another perspective, due to concerns about the physical spread of the virus across state boundaries that results from the interactions of geographically proximate populations (Macinko & Silver, 2015), a governor may see a SAHO in a neighboring state as a signal of the severity of the threat in that state and feel the need to also issue a SAHO to protect her or his state’s residents. Although the research is mixed, it is reasonable to expect that *governors in states with more geographically proximate states with SAHOs will be more likely to issue a SAHO*.

Finally, policies may temporally diffuse across states in a systematic manner. For instance, Berry (1999) report that policies may spread across states following an S-shaped curve in which a small number of leading states adopt a policy before most others (see also Mooney & Lee., 1995). This gives the remaining states the opportunity to evaluate the efficacy of the policy and how citizens respond to it. As with geographic diffusion, this period of observation allows these states to learn from the adoptions of leader states, which reduces the risk of adoption and leads to an increasing rate of subsequent adoptions. Finally, a smaller number of laggard states adopt. This suggests it is reasonable to expect that *governors will be more likely to issue a SAHO over time following an S-shaped temporal pattern*.

### Data and methods

This research uses event history analysis (EHA) to estimate the effects of scientific, political, social, economic, and external factors on the probability a governor will issue a COVID-19-related SAHO. EHA is a time-series technique used to estimate relationships with rare events and is commonly used in policy adoption research (Berry & Berry, 1992). In particular, it captures the timing, number, and sequence of events in a process to understand the effect of time in that process and, more broadly, to identify patterns of change and factors contributing to change in that process (e.g., Box-Steffensmeier and Jones, 1997). It uses panel data consisting of non-repeatable events to identify the association between, in this case, the gubernatorial issuance of a COVID-19-related SAHO and internal state factors in policy adoption and external factors of policy diffusion.

Because the dependent variable is dichotomous, the relationship between issuance of a SAHO and the factors are estimated using probit regression. Diagnostic tests present evidence of heteroskedasticity, so the model is estimated using robust standard errors. EHA assumes the beginning and ending points of data collection are theoretically motivated (Box-Steffensmeier and Jones, 1997). The time frame assessed includes the 41 days from March 1 to April 10, 2020. March 1 is the day after the first coronavirus-related death in the U.S. was reported and the first governor declared a coronavirus-related state of emergency (both in Washington). These unambiguous events indicate a reasonable date for what is frequently identified as the first stage in the policy process, problem identification, and, therefore, suggest a date from which to collect and analyze data. March 14, which is the day following two national emergency declarations by President Trump (National Conference of State Legislatures, 2020), is also a reasonable starting date. Analyses using a time frame starting on March 14 results in substantively similar results.^2^ On the other hand, April 10 is shortly after the last SAHO was issued on April 7 (in South Carolina) and a reasonable date to end data collection.

We construct the dependent variable of gubernatorial issuance of a SAHO or not from official sources (e.g., statements from governors’ offices or tweets from governors) informed by news reports from the *New York Times* (Mervosh et al., 2020; see Appendix 1). As with survival analysis, a subject (state) drops out of the analysis once it is no longer at risk of adopting the policy (once the governor has issued the order). For example, the New York SAHO went into effect on March 22. It was coded 0 for the 21 observations from March 1 to March 21, then 1 on the one observation on March 22, the day on which the state’s SAHO went into effect, and subsequently omitted from March 23 to April 10. When capturing all 50 states, this results in 1483 state-day observations.

We gathered the data on the independent variables from secondary sources such as the U.S. Census Bureau, the National Conference of State Legislatures, and the *Washington Post* (Fox et al., 2020). The analyses also control for media coverage following Walgrave and van Aelst (2006). Media coverage can stimulate public concern about an issue that, in turn, motivates political elites to address the issue. On the other hand, the media are more likely to cover an issue that already has the attention of policymakers (Kingdon, 1995). This suggests the possibility of simultaneity, which is addressed in the data by lagging the media coverage variable. See Table 1 for descriptions and additional details on the variables.

**Table 1.** Descriptive statistics and sources for variables.

		*Variable*	*Mean*	*Std. Dev*	*Min/Max*	N^*^	*Description [Source]*
	Status (DV)		0.0	0.2	0/1	1483	Gubernatorial issuance of a SAHO (coded 1) or not (coded 0) on a given state in a given day [official sources]
1	Deaths/Bed		1.6	5.2	0/62.7	1483	Number of cumulative deaths (lagged 1 day) per intensive care unit bed per 10,000 population [Fox et al., 2020; kaiser family Foundation’s state health facts, 2019]
2	Population65+		16.5	1.9	11.1/20.6	50	Percentage of population age 65 and older [U.S. Census Bureau, 2019]
3	DemGov		0.5	0.5	0/1	50	Political party of the governor is democratic (coded 1) or not (coded 0) [National Conference of State Legislatures, 2019]
4	DemElect		47.0	11.0	24.3/67.4	50	Percentage of the two-party vote in the 2016 presidential election received by the democratic candidate [U.S. Federal Election Commission, 2017]
5	Collectivism		50.1	11.3	31.0/91.0	50	Measure of collectivist versus individualistic tendencies [Vandello & Cohen, 1999]
6	Unemployment		3.6	0.8	2.4/6.1	50	Number of eligible workers who are unemployed as a percent of the labor force in 2019 [U.S. Bureau of labor statistics -- department of Labor, 2019]
7	GeoDiffusion		0.1	0.2	0/1	1483	Proportion of contiguous states that have previously issued a SAHO (lagged 1 day) [author generated statistic based on Chamberlain & Haider-Markel, 2005]
8	TempDiffusion		4.1	1.3	0/5.7	1483	Square root of the number of days between a given state-day and the day with the highest order rate or hazard rate, in this case April 3 (see Appendix 1) [author generated statistic based on Mooney & Lee., 1995]
-	Media		87.5	150.5	0/1058	1483	Cumulative state newspaper story counts (lagged 7 days) where the entry has the document type indicated as “news” and includes the terms “COVID-19,” “coronavirus,” or “pandemic” [ProQuest U.S. Newsstream]

Proportions are presented for indicator variables.

*N=50 when the mean for the 50 states is used because the variable is constant throughout the study period; N=1483 when the mean for the 1483 cases is used because the variable changes through the study period.

## Results

For transparency to allow readers to assess the basic associations between the dependent and independent variables for considerations of relationship robustness, suppression effects, and confounding (Lenz & Alexander, 2021), Appendix 2 reports nine bivariate probit regression models, one for each independent variable and the dependent variable. Each model includes 1483 state-day observations. Model fit is statistically significant in six of the nine models. McFadden’s pseudo *R*^2^ ranges from 0.00 to 0.18. The models correctly classify 97.2% of the cases with proportional reduction in error (PRE) ranging from 0.0 to 2.4. Bayesian Information Criterion (BIC) ranges from 329.7 to 396.2. Of most import, the relationships are mostly robust to multivariate versus bivariate specification. Only one variable (unemployment) switches from statistical insignificance in the bivariate model to statistical significance in the full model, which may indicate a suppression effect, and one (media) switches from statistical significance in the bivariate model to statistical insignificance in the full model, which may indicate confounding.

Appendix 3 reports the full probit regression model. The number of observations is 1483 (state-days). The overall model fit is statistically significant (Log pseudolikelihood = −131.2, LR [9] = 118.9, *p* < 0.001) with a McFadden’s pseudo *R*^2^ of 0.31. The model correctly classifies 97.6% of the cases with a proportional reduction in error of 14.3%. There is little to no evidence that multicollinearity threatens the results. No pair of variables reaches the common problematic threshold of a bivariate correlation of 0.9. Only two pairs exceed 0.5: DEMgov and demVote (r=0.51) and geoDIFF and tempDIFF (r=0.69). Further, no individual variable carries a variance inflation factor (VIF) value of greater than 10 (the maximum is 2.33 for tempDiff), and the mean VIF is well less than the problematic threshold of 6. No tolerance is less than 0.1, and the maximum *R*^2^ is 0.57 (tempDiff).

Because probit estimates provide limited intuitive information beyond direction of effect and statistical significance, Table 2 reports the results in terms of the estimated marginal effect of each independent variable on the probability that a governor will issue a COVID-19-related SAHO on any given day as derived from the probit estimates.

**Table 2.** Effects on gubernatorial stay-at-home orders.

		Unstandardized		Min_x_/Max_x_		
	Variables (Expected Sign)	Marginal Effects	95% CI	Marginal Effects	95% CI	Expected Effect
1	*Deaths/Bed* (+)	0.001	(0.000, 0.002)*	0.053	(0.005, 0.100)*	Yes
2	*Population65+* (+)	0.002	(−0.003, 0.006)	0.015	(−0.029, 0.060)	No
3	*DemGov* (+)	0.025	(0.002, 0.048)*	0.025	(0.002, 0.048)*	Yes
4	*DemElect* (+)	0.002	(0.000, 0.003)*	0.076	(0.018, 0.134)*	Yes
5	*Collectivism* (+)	−0.000	(−0.001, 0.001)	−0.006	(−0.050, 0.038)	No
6	*Unemployment* (−)	0.015	(0.004, 0.026)*	0.055	(0.014, 0.097)*	No
7	*GeoDiffusion* (+)	0.031	(−0.001, 0.063)‡	0.031	(−0.001, 0.063)‡	No
8	*TempDiffusion* (−)	−0.026	(−0.038, −0.015)*	−0.151	(−0.216, −0.086)*	Yes
-	*Media*	−0.000	(−0.000, 0.000)	−0.025	(−0.063, 0.013)	

* *p* < 0.05, ‡ *p* < 0.10 (two-tailed test).

CI = confidence interval.

The results suggest that science was a factor in governors’ decisions to issue a SAHO. The first expectation is that considerations of “flattening the curve” influenced the issuance of SAHOs. The independent variable, *Deaths/Bed*, captures the lagged (1 day) number of cumulative COVID-19-related deaths per ICU bed per 10,000 state population. Table 2 reports that each additional death per ICU bed per 10,000 state population is associated with a statistically significant marginal effect of one-tenth percentage-point increase in the probability that a governor would issue a SAHO on any given day. This translates into a statistically significant 5.3-percentage-point increase in probability when a state moves from the minimum (0) to the maximum (62.7) number of deaths per ICU bed during the time of the study. This result is robust to alternative measures (this and other robustness check models are not reported here) of confirmed cases per ICU bed, deaths and confirmed cases per standard hospital bed per 10,000 population (Kaiser Family Foundation, 2019), and deaths per medical doctor per 100,000 population from the Association of American Medical Colleges (2019).

The results, though, do not support the second scientific expectation, which is that population vulnerability would play a role. The independent variable *Population65+* indicates the percentage of population 65 years old and older in a state. Table 2 shows the effect is not statistically meaningful. This null finding is confirmed by alternative specifications using a measure of general state health scores produced by the Kaiser Family Foundation (2019) in the form of a health ranking analysis based on a comprehensive set of health, environmental, and socioeconomic measures as well as percent urban population and population density from the U.S. Census Bureau.

The results strongly suggest that political considerations were a factor in governors’ decisions to issue a SAHO. The expectations are that Democratic governors are more likely to issue a COVID-19-related SAHO than Republican governors as are governors in states where a greater proportion of the electorate leans toward the Democratic Party. The independent variable *DemGov* is an indicator variable coded 1 for states with a Democratic governor and 0 when a Republican governor. All 24 Democratic governors issued a SAHO, while 18 (69%) of the Republican governors did. The independent variable, *DemElect*, indicates the percentage of the two-party vote that the Democratic presidential candidate received in the 2016 election. Table 2 indicates that Democratic governors are a statistically significant 2.5 percentage points more likely to issue a SAHO than Republican governors on any given day. Further, each one-percentage-point increase in Democratic vote share in 2016 is associated with a statistically significant two-tenths percentage-point increase in the probability that a governor will issue a SAHO on any given day. While the effect of *DemGov* does not change due to it being an indicator variable, Table 2 indicates there is a 7.6-percentage-point change when a state moves from the minimum percentage of Democratic voters (24.3) to the maximum percentage (67.4).

On the other hand, there is no meaningful evidence that social considerations were a factor in governors’ decisions regarding SAHOs. The expectation is that states with more collectivistic (vs. individualistic) civic cultures, as represented by the independent variable *Collectivism,* are more likely to support a SAHO. This measure is based on eight items, the first three of which are related to state residents’ family structure and living arrangements and the remaining items are related to residents’ social, economic, religious, and political practices (Vandello & Cohen, 1999). Table 2 shows that the effect is not meaningful. This result is confirmed by an alternative specification using Rice and Sumberg’s (1997) measure of civic mindedness based on levels of civic cooperation; civic engagement; political equality; and solidarity, trust, and tolerance.

The results also indicate that economic considerations were a factor. Although contrary to expectations, when states’ economies are weaker as indicated by increased unemployment, the probability that governors issue a SAHO increases. Unemployment is indicated by the variable *Unemployment*. Table 2 indicates that each one-percentage-point increase in unemployment increases the probability of a SAHO by a statistically significant 1.5 percentage points on any given day. The change in probability increases by 5.5 percentage points when a state moves from the minimum (2.4) to the maximum (6.1) rate. This statistically significant result is robust to an alternative specification using the Standard and Poor’s State Rating (Standard & Poor’s Global Market Intelligence, 2020), which is a highly referenced estimate of the ability of states to repay their debt obligations or avoid default.

**Figure 1.**
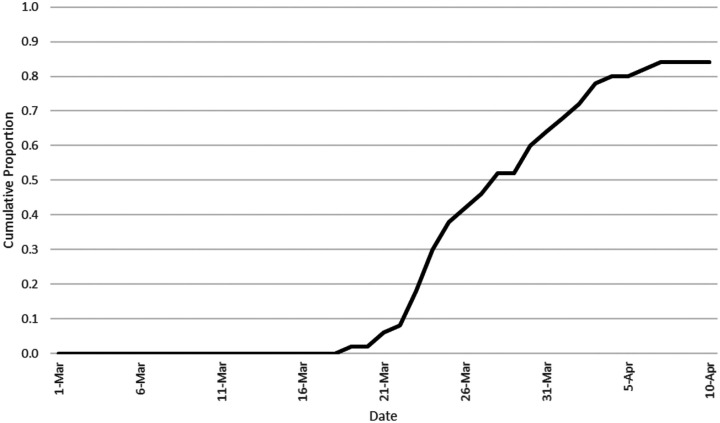
Temporal diffusion as indicated by the cumulative proportion of states with SAHOs.

Finally, external factors also played a role in governors’ decisions to issue SAHOs. One expectation is that geographic diffusion, as represented by *GeoDiffusion*, should positively affect governors’ decisions to issue a SAHO. Table 2 indicates the effect approaches but does not reach conventional levels of statistical significance. Further analysis indicates an alternative specification using the U.S. Department of Commerce’s Bureau of Economic Analysis (BEA) regions, which groups states into eight regions based on their social and economic homogeneity, fails to reach even the marginal level of statistical significance achieved by *GeoDiffusion*. These results offer weak support at best for this expectation. A second expectation is that temporal diffusion, as represented by *TempDiffusion*, should also affect governors’ decisions, and because of the way it is constructed the sign should be negative (Mooney & Lee., 1995; see Table 1 for description). Figure 1 depicts the S-shaped temporal pattern and Table 2 reports a statistically significant change in the probability of a SAHO following an S-shaped diffusion pattern such that a time period of slow adoption is followed by a time period of accelerated adoption which is followed by another period of slow adoption.

For complete reporting, note that Table 2 also shows the control variable media coverage, *Media*, is not statistically related to the issuance of SAHOs.

### Assessing the Relative Effects of the Individual Considerations

**Figure 2.**
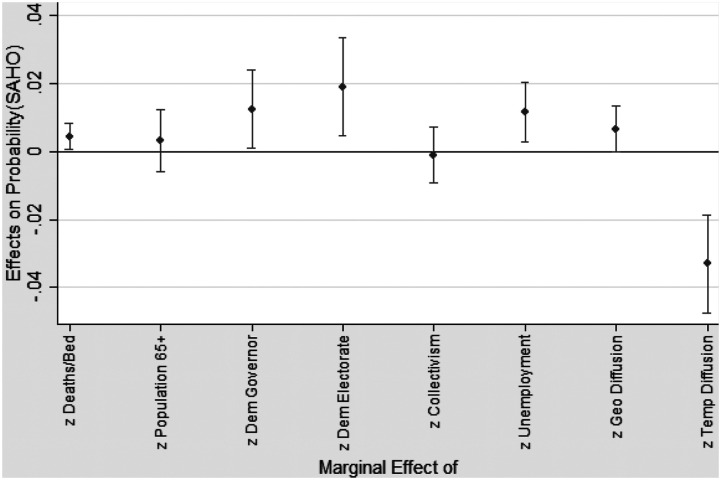
Change in probability of a SAHO based on a 1 standard deviation change in each independent variable (95% Confidence Intervals).

Calls for evidence-based policy making suggest that rigorously established objective evidence should play a primary role in policy making (e.g., Brownson et al., 2017). Figure 2 depicts the change in probability of a SAHO on any given day based on a one-standard-deviation change in each independent variable. This analysis permits direct comparison of the importance of each measure, in particular a comparison of the relative effect of the scientific considerations to the political, social, economic, and external considerations. The marginal effect of Democratic vote share (*z_DemElect*: dy/dx = 0.019, 95% CI = 0.005, 0.033) is nearly five times the effect of deaths per ICU bed (*z_Deaths/Bed*: dy/dx = 0.004, 95% CI = 0.00, 0.008), while the effect of a Democratic governor (*z_DemGov*: dy/dx = 0.012, 95% CI = 0.001, 0.024) is three times the effect of deaths per ICU bed. Similarly, the effect of economic status (*z_Unemployment*: dy/dx = 0.012, 95% CI = 0.003, 0.020) is three times the effect of deaths per ICU bed. While geographic diffusion is similar in effect (*z_GeoDiffusion*: dy/dx = 0.007, 95% CI = −0.000, 0.013), most notably temporal diffusion has approximately eight times the effect (*z_TempDiffusion*: dy/dx = −0.033, 95% CI = −0.047, −0.019). These results suggest the scientific factors, which some may characterize as evidence-based measures, did not play the important role called for by proponents of evidence-based policy making.

### Assessing the Relative Effects of the Five Broad Factors

It is also useful to compare the effects of the broad factors. Table 3 presents several analyses that indicate the relative importance of the five factors. The first column of results reports post-estimation tests of the joint significance of the variables that comprise each factor. For example, the analysis of the political factor includes the Democratic governor and Democratic electorate variables. These Wald tests indicate that the external, political, and economic factors create statistically significant improvements in the fit of the model at conventional levels of statistical significance, while the scientific factor does so at only a marginal level of statistical significance (*p* = 0.098 two tailed). The social factor has no discernible statistical effect.

**Table 3.** Relative Effects of the Broad Factors.

	Joint Significance	Factor Excluded		
Factor	Wald χ^2^	BIC	PRE (%)	Pseudo *R*^2^
External	63.31*	**396.73**	**2.38**	**0.12**
Political	14.24*	349.64	7.14	0.24
Economic	7.29*	335.26	4.76	0.29
Scientific	4.64+	*324.26*	9.52	0.30
Social	*0.07*	328.17	*14.29*	*0.31*

* *p* < 0.05 + *p* < 0.10 two tailed.

Most important factor **bolded**; least important factor *italicized*.

The remaining columns report analyses in which the variables for the specified factor are excluded from the full model. For instance, the analyses reported for the political factor include all the variables except Democratic governor and Democratic electorate. The Bayesian Information Criterion (BIC) is used to rank a group of models based on model uncertainty (Raftery, 1995). In particular, the model with the lowest BIC score is the most desirable model, because it demonstrates the least uncertainty related to out-of-sample prediction. Raftery (1995: 139) suggests that between-model BIC score differences of 0–2 provide “weak” evidence for preferring one model over another, 2–6 provide “positive” evidence, 6–10 provide “strong” evidence, and differences of 10 or more provide “very strong” evidence. Based on these guidelines, Table 3 provides “very strong” evidence that the model that excludes the external factor (geographic and temporal diffusion) creates the most uncertainty and is the “worst” model by far. On the other hand, the model that excludes the scientific factor (deaths per ICU bed and population vulnerability) creates the least uncertainty and is the “best” model. This suggests that the external considerations played a much larger role and were much more important in governors’ decisions to issue SAHOs than scientific considerations. Alternatively, the estimates of PRE and pseudo *R*^2^ also suggest that external considerations played the largest and most important role by also indicating that excluding them produces the least desirable model as indicated by the smallest PRE and pseudo *R*^2^. They diverge from BIC, though, by suggesting that the least important factor is the social factor, with the scientific considerations a distant second in terms of PRE. In all, the combined evidence in Table 3 suggests that the external factor played a substantially greater role in governors’ decisions to issue SAHOs than the scientific factor and the political factor likely played a greater role as well.

## Discussion and conclusion

Issuing a stay-at-home order is among the most consequential acts a U.S. state governor can take. While these orders protect public health, they also impose substantial social and economic costs on their states’ residents (e.g., Curley & Federman, 2020; Gostin & Wiley, 2020; Pew Research Center, 2020). The vast majority of governors issued such orders, but they did it at different times, and eight of the 50 did not issue one at all (Mervosh et al., 2020). This study is intended to identify scientific, political, social, economic, and external factors that may have influenced governors to make the profound and high-stakes decision to issue SAHOs to slow the spread of the first wave of COVID-19. The findings suggest that scientific factors concerning “flattening the curve” influenced governors’ decisions (governors in states with a greater incidence of the disease relative to healthcare capacity were more likely to issue a SAHO) but not related to concerns over a greater proportion of vulnerable populations.

The results in Table 3, though, indicate that external considerations played the largest role by far. The decisions of neighboring states played a role, although the timing of adoptions in other states seemingly mattered the most, with more than half of all the SAHOs issued over a 6-day period (March 23–28). The data depicted in Figure 1 clearly reflect the S-shaped temporal adoption pattern noted by Berry (1999). Political factors related to the partisanship of the governor (Democratic governors were more likely to issue a SAHO) and the electorate (governors in states with more Democratic voters were more likely to issue a SAHO) played the next largest role. Economic factors related to the health of the economy (governors of states with stronger economies were less likely to issue a SAHO) played a much lesser role, while it appears social factors had little to no effect.

Relatively speaking, the results depicted in Figure 2 and reported in Table 3 indicate that scientific considerations played a limited role, particularly compared to the external and political factors. As Spasoff (1999) noted, political actors play an important role in public health policy, and even well-intentioned elected officials are likely to view facts and decisions through a political lens (Mayhew, 1974). The relative effects, then, may not come as a surprise. Despite calls for evidence-based policy making (e.g., Brownson et al., 2017) and aspirations for elected officials to act as trustees for the broader public interest (Burke, 1790), it should not be surprising that they may act as delegates representing political interest among their constituents, for scientific good or bad. From a very broad perspective, one might argue that in a democracy political attitudes should play a meaningful role along with other important factors like scientific considerations.

It is worth noting several limitations of this study. This is one examination of one rare but urgent event that happened over a very short period of time. Further, these are observational data, which imply correlations but cannot establish causation. One should generalize with great caution, if at all. These results reflect the analysis of publicly available data; they do not and cannot indicate the effects of non-public data to which governors have access or governors’ private motivations or fears. These details most likely will be uncovered as the coronavirus threat plays out over time and further research is undertaken. Given the sensitivities of curtailing people’s liberties in the U.S., it is not certain that governors could comfortably forecast how their residents would react to these mandates. There is no guarantee of governors’ intentions to enforce their SAHOs (Norwood, 2020). Finally, the study does not consider the effects of local policies, which may serve as an impetus to state action (Quinton, 2017). This leaves open an important question about how local policies may have informed state action.

In conclusion, this study offers evidence about how political leaders prioritized science and other factors in their early response to the COVID-19 pandemic. As a result of their responses, more than 90% of the U.S. population was affected by governors’ decisions to issue scientifically effective but socially and economically costly SAHOs (Norwood, 2020). This investigation suggests that these decisions were affected by multiple factors and, in particular, indicate that science was a low priority relative to external and political factors. Early investigations like this have an important role to play. This study offers to individuals, communities, organizations, policy makers, and researchers information pertinent to understanding far-reaching government responses to large-scale crises in general and healthcare crises more specifically.

## Notes

1. Additionally, some also may calculate that this will negatively impact her or his vote share in the next election (Niemi et al., 1995).

2. While the results indicate weaker model fit, the associations between the individual independent variables and dependent variable are only trivially different and yield no change in levels of statistical significance.

## Appendix 1

Daily hazard rates for states issuing a stay-at-home order

**Table:**

Date (2020)	Declarations^a^	Risk set^b^	Hazard rate^c^, %	States^*^
1-18 March	0	50	0	
19 March	1	50	2	California
20 March	0	49	0	
21 March	2	49	4	Illinois, New Jersey
22 March	1	47	2	New York
23 March	5	46	11	Connecticut, Louisiana, *Ohio*, Oregon, Washington
24 March	6	41	15	Delaware, *Indiana*, *Massachusetts*, Michigan, New Mexico, *West Virginia*
25 March	4	35	11	Hawaii, *Idaho*, *Vermont*, Wisconsin
26 March	2	31	6	Colorado, Kentucky
27 March	2	29	7	Minnesota, *New Hampshire*
28 March	3	27	11	*Alaska*, Montana, Rhode Island
29 March	0	24	0	
30 March	4	24	17	Kansas, *Maryland*, North Carolina, Virginia
31 March	2	20	10	*Arizona*, *Tennessee*
1 April	2	18	11	Nevada, Pennsylvania
2 April	2	16	13	Maine, *Texas*
3 April	3	14	21	*Florida*, *Georgia*, *Mississippi*
4 April	1	11	9	*Alabama*
5 April	0	10	0	
6 April	1	10	10	*Missouri*
7 April	1	9	11	*South Carolina*
8-10 April	0	9	11	

^*^ States names with Republican governors are italicized.

No orders issued as of April 10, 2020: Arkansas, Iowa, Nebraska, North Dakota, Oklahoma, South Dakota, Utah, Wyoming.

^a^Declarations = number of stay-at-home orders (SAHO) issued on a given day.

^b^Risk Set = total number of states not previously having passed a SAHO.

^c^Hazard Rate = Declarations/Risk Set.

## Appendix 2

Bivariate Probit Regression Models

**Table:**

		(1)	(2)	(3)	(4)	(5)	(6)	(7)	(8)	(9)
(1)	*Deaths/Bed*	0.047^*^								
		(0.007)								
(2)	*Population 65+*		0.026							
			(0.035)							
(3)	*DemGov*			0.273^*^						
				(0.134)						
(4)	*DemElect*				0.015^*^					
					(0.006)					
(5)	*Collectivism*					0.006				
						(0.005)				
(6)	*Unemployment*						0.109			
							(0.077)			
(7)	*GeoDiffusion*							1.706^*^		
								(0.203)		
(8)	*TempDiffusion*								0.428^*^	
									(0.040)	
(9)	*Media*									0.001^*^
										(0.000)
	*_cons*	−2.061^*^	−2.343^*^	−2.037^*^	−2.620^*^	−2.212^*^	−2.296^*^	−2.233^*^	−0.464^*^	−2.061^*^
		(0.072)	(0.581)	(0.097)	(0.294)	(0.282)	(0.288)	(0.085)	(0.152)	(0.076)
	*N*	1483	1483	1483	1483	1483	1483	1483	1483	1483
	Pseudo *R*^2^	0.09	0.00	0.01	0.02	0.00	0.00	0.14	0.18	0.04
	*BIC*	361.1	396.2	392.6	391	395.7	395.1	343.6	329.7	381.9
	*CC%*	97.2	97.2	97.2	97.2	97.2	97.2	97.2	97.2	97.2
	*PRE%*	2.4	0.0	0.0	0.0	0.0	0.0	0.0	0.0	0.0
	Log likelihood	−173.2^*^	−190.8	−189.0^*^	−188.2^*^	−190.6	−190.2	−164.5^*^	−157.5^*^	−183.6^*^

Coefficient (standard error). * *p* < 0.05 + *p* < 0.10 two tailed. CC% = correctly classified %.

## Appendix 3

Multivariate Regression Model

**Table:**

	Variables (Expected Sign)	Coefficient	95% CI
1	*Deaths/Bed* (+)	0.018	(0.002, 0.035)*
2	*Population65+* (+)	0.035	(−0.066, 0.136)
3	*DemGov* (+)	0.542	(0.040, 1.045)*
4	*DemElect* (+)	0.038	(0.009, 0.067)*
5	*Collectivism* (+)	−0.002	(−0.018, 0.014)
6	*Unemployment* (−)	0.323	(0.088, 0.557)*
7	*GeoDiffusion* (+)	0.672	(0.003, 1.342)*
8	*TempDiffusion* (−)	−0.567	(−0.794, −0.339)*
-	*Media*	−0.001	(−0.001, 0.000)
	*_cons*	−3.922	(−6.120, −1.723)*
	*N*	1483	
	Pseudo *R*^2^	0.31	
	*BIC*	335.39	
	*Correctly Class%*	97.6%	
	*PRE%*	14.3%	
	Log likelihood	−131.2	

* *p* < 0.05, ‡ *p* < 0.10 (two-tailed test).

CI = confidence interval; CC% = correctly classified %.
